# Multi-Segment Direct Inject nano-ESI-LTQ-FT-ICR-MS/MS For Protein Identification

**DOI:** 10.1186/1477-5956-9-38

**Published:** 2011-07-07

**Authors:** Jing Chen, Lorena Canales, Rachel E Neal

**Affiliations:** 1 Dept of Environmental and Occupational Health Sciences, School of Public Health and Information Sciences, University of Louisville, 485 E. Gray Street, Louisville, KY 40202, USA

**Keywords:** Direct inject, Nano-ESI, LTQ-FT-ICR-MS/MS, Protein identification

## Abstract

Reversed phase high performance liquid chromatography (HPLC) interfaced to electrospray tandem mass spectrometry (MS/MS) is commonly used for the identification of peptides from proteolytically cleaved proteins embedded in a polyacrylamide gel matrix as well as for metabolomics screening. HPLC separations are time consuming (30-60 min average), costly (columns and mobile phase reagents), and carry the risk of column carry over between samples. The use of a chip-based nano-ESI platform (Advion NanoMate) based on replaceable nano-tips for sample introduction eliminates sample cross-contamination, provides unchanging sample matrix, and enhances spray stability with attendant increases in reproducibility. Recent papers have established direct infusion nano-ESI-MS/MS utilizing the NanoMate for protein identification of gel spots based on full range MS scans with data dependent MS/MS. In a full range scan, discontinuous ion suppression due to sample matrix can impair identification of putative mass features of interest in both the proteomic and metabolomic workflows. In the current study, an extension of an established direct inject nano-ESI-MS/MS method is described that utilizes the mass filtering capability of an ion-trap for ion packet separation into four narrow mass ranges (50 amu overlap) with segment specific dynamic data dependent peak inclusion for MS/MS fragmentation (total acquisition time of 3 minutes). Comparison of this method with a more traditional nanoLC-MS/MS based protocol utilizing solvent/sample stream splitting to achieve nanoflow demonstrated comparable results for protein identification from polyacrylamide gel matrices. The advantages of this method include full automation, lack of cross-contamination, low cost, and high throughput.

## Background

One of the major goals of proteomics is to be able to identify proteins of interest [1]. For protein spots on 1D- or 2D-SDS-PAGE gels this is a challenge due to the large diversity of protein spot abundances and the complexity of the matrix. The great number of protein spots or gel slices which can approach several hundred per experiment demands automated, high throughput, cost-effective analytical methods with high sensitivity [2].

For the proteomics workflow, high performance liquid chromatography (HPLC) is typically coupled via an electrospray source (nanoESI or ESI) to either an ion trap or time-of-flight (TOF) mass analyzer with ion packet selection via mass filter (quadrapole or ion trap) [2-9]. Following parent mass feature selection, tandem mass spectrometry (MS/MS) based on collision induced dissociation (CID) or electron capture dissociation (ECD) of peptide parent mass/charge features are used to generate mixed ion populations of peptide fragments which are then utilized for protein identification. The acidified HPLC eluent serves two purposes: a) to separate peptide mixtures thus simplifying the parent mass composition of ion packet; and b) to competitively eliminate salts. However this workflow has several drawbacks including potential sample cross-contamination due to column carryover and the lengthy sample run time [2,8]. The cost of instrument time, columns, and solvents drives the total cost per sample.

To address the problem of lengthy sample run time and expensive instrument time usage, direct infusion sample introduction methods without HPLC separation prior to the electrospray source has been developed [2,8,10-16]. The advantages of the chip-based ESI method include fully automated, high throughput, a lack of cross-contamination, enhanced spray stability and reproducibility, and constant sample matrix [2,8,13-16]. In each case, protein spots were analyzed with one broad mass range MS scan followed by MS/MS scans.

In the current study, a method of direct inject LTQ-FT-ICR-MS/MS combined with automated chip-based nanoESI is introduced which is broadly applicable to a variety of mass spectrometry types. Proteolytic peptides were subjected to a MS prescan (00~2000 m/z) followed by four narrow mass range scans with 50 amu overlap and including iterative MS/MS of mass features in the selected window with total acquisition time of 3 min per sample. Dynamic data dependent exclusion and charge state screen were enabled in these ranges to ensure that many +2 and +3 charged peaks were selected based on ion intensity. This new multi-segment direct inject nano-ESI method was compared to a traditional RPLC-nanoESI-LTQ-FT-ICR-MS/MS method with an approximate run time of ~30 minutes. The ability to identify proteins from rat liver 2D-SDS-PAGE gel spots was compared between methods.

## Results

**Figure 1:**
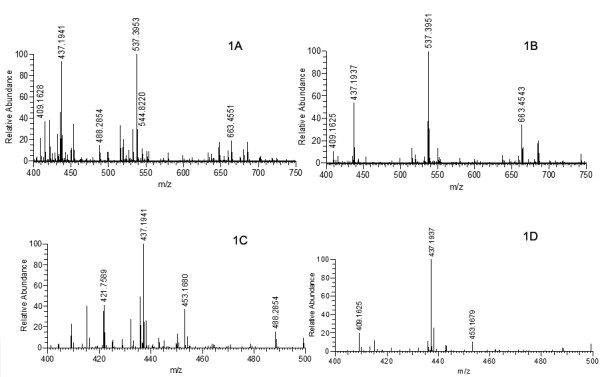
**LTQ-FT-ICR-MS spectra of tryptic digests from a single gel spot scanned across different m/z ranges**. **Fig. 1A**, Tryptic digest sample scanned from 400-750 m/z range. **Fig. 1B**, same tryptic digest sample scanned from 400-2000 m/z range, spectrum shown zoomed to 400-750 m/z. **Fig. 1C & 1D**, spectra of A and B zoomed to the range of 400-500 m/z.

As shown in Figure 1, scanning across different mass ranges impacts the number and intensity of the parent ion LTQ-FT-ICR-MS spectra generated from tryptic digests of protein spots. In Figure 1A, a selected segment from the multi-step method (400-750 m/z) is shown. In Figure 1B, a scan of the full experimental mass range was collected (400-2000 m/z) with a zoom to the mass region comparable to that shown in Figure 1A (400-750 m/z). Clear differences in the number and intensity of ions present in the spectra are found with the sole difference between spectra being mass range selection of ion packet--this highlights the impact of discontinuous ion suppression and ion population characteristics on spectral features. Specifically, the loss of numerous moderate to low abundance ion features is evident in Figure 1B (mass range scanned 400-2000 m/z). Figure 1C &1D are identical spectra from Figure 1A and 1B but zoomed to the range of 400-500, respectively, for enhanced comparison with the identical outcome of a greater number of ion features present in the narrow scan range (400-750 m/z; Figure 1C) versus the broad ion scan (400-2000 m/z; Figure 1D). A greater number of +2 and +3 charged ions were assigned (not identified for simplicity) when the smaller range was scanned further highlighting the utility of this method for iterative data dependent MS/MS analysis.

**Figure 2:**
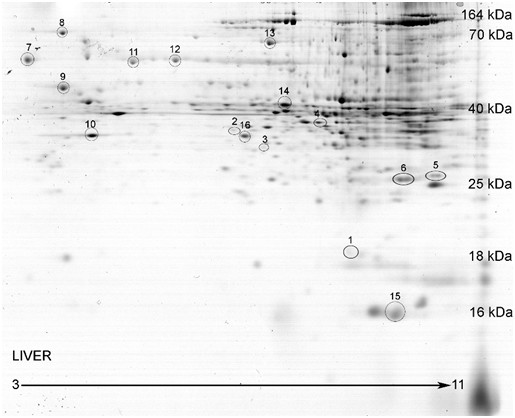
**Representative image of mouse liver proteins separated by 2D SDS-PAGE**. The protein spot density of each spot was normalized to the total spot density of all spots on the gel. The proteins on the gel span an isoelectric focusing point of 3 to 11. The acidic proteins are on the left, while the basic proteins are on the right. The molecular weights of the liver protein spots are within the range of approximately 10 kDa and 170 kDa. Several proteins spots were identified to reference the molecular weights listed along the gel. There are varying intensities in the proteins spots on the gel image. The darker spots (or spots that are higher in intensity) are more abundant protein spots, while the lower intensity spots are lower abundance proteins.

In Table 1 (Figure 2 liver gel image with spot numbering), the ability to identify proteins from 16 protein containing gel spots by these two methods were compared. A total of 41 proteins were identified by HPLC-MS/MS method while 38 proteins were identified by chip-based nanoESI/MS/MS method. This reflects the known outcome of multiple proteins present in protein spots from 2D gels. A total of 33 common proteins were identified with both methods. Twenty out of the 33 common proteins identified by both methods possess higher MOWSE scores with the multistep direct inject nanoESI/MS/MS method. For the remaining 13 common proteins, the MOWSE scores are comparable between methods. Interestingly a lack of putative identity hits from decoy database searching (reverse amino acid sequence for proteins used to assess false positive identifications) was observed for the multistep nanoESI/MS/MS method. However, when utilizing the LC-based separation method, spurious putative identifications were present. A larger study will be necessary to confirm that the multistep method yields higher identification certainty with lower false-positive rate. Of note, the multistep direct inject method yielded a greater number of peptides matched per protein and a higher percent coverage for the majority of the common proteins identified by both methods (22 out of 33). For the remainder, 6 possess equal scores and 5 are of comparable ranking.

**Table 1:** Comparison of identification of 2D gel separated spots of rat liver protein by chip-based nanoESI/MS/MS and nanoHPLC-MS/MS

Spot	Method	Protein	Score	Peptidesmatched	% Cover	ProteinMW (× 10^-3^)	Decoy Discovery Rate	False Discovery Rate	Accession
1	HPLC	Protein disulfide-isomerase	211	7	12	57.1	0	0	gi129729

	Multistep		280	15	26		0	0	

	HPLC	Iodothyronine 5' monodeiodinase	211	7	13	54.0			gi202549

	Multistep		280	15	25				

	HPLC	Prolyl 4-hydroxylase, beta polypeptide, isoform CRA_a	211	7	11	59.0			gi148702818

	Multistep		280	15	25				

	HPLC	Prolyl 4-hydroxylase, beta polypeptide, isoform CRA_b	211	7	11	61.5			gi148702819

	Multistep		280	15	24				

2	HPLC	Methionine adenosyltrans-ferase I, alpha	304	7	21	43.5	0	0	gi19526790

	Multistep		378	6	28		0	0	

3	HPLC	Regucalcin	230	5	18	33.4	0	0	gi6677739

	Multistep		211	4	14		0	0	

4	HPLC	Carbamoyl-phosphate synthetase 1	1286	28	18	164.5	4	17.39	gi124248512

	Multistep		697	23	14		0	0	

5	HPLC	Carbamoyl-phosphate synthetase 1	1221	27	18	164.5	3	17.65	gi124248512

	Multistep		1112	31	20		0	0	

6	HPLC	Glutamate de-hydrogenase 1	416	9	17	61.3	1	9.09	gi6680027

	Multistep		364	10	17		0	0	

7	HPLC	Arginase 1	284	5	16	34.8	1	12.5	gi7106255

	Multistep		290	8	26		0	0	

	HPLC	Short-chain specific acyl-CoA dehydrogenase, mitochondrial	134	4	10	44.9			gi584714

	Multistep		192	6	19				

8	HPLC	Sorbitol dehydrogenase precursor	176	4	11	40.7	0	0	gi1009706

	Multistep		207	6	17		0	0	

	HPLC	Sorbitol dehydrogenase	176	4	12	38.2			gi22128627

	Multistep		207	6	18				

	HPLC	L-iditol 2-dehydrogenase	176	4	11	42.8			gi397357

	Multistep	Sorbitol dehydrogenase, isoform CRA_a	176	4	14	32.0			gi149023127

9	HPLC	Fumaryl-acetoacetase	191	4	10	46.2	0	0	gi50973

	Multistep		290	6	15		0	0	

	HPLC	4-hydroxy-phenyl-pyruvate dioxygenase	197	4	10	45.1			gi849053

	Multistep	F1 protein	197	4	10	43.6			gi1841443

	HPLC	Chain A, Crystal Structure of Fumarylacetoacetate Hydrolase Complexed W/4-(Hydroxymethylphosphinoyl)-3-Oxo-Butanoic Acid	191	4	10	46.2			gi13399972

	Multistep	Chain A, Crystal Structure of Fumarylacetoacetate Hydrolase	191	4	10	45.9			gi8569272

	HPLC	Chain A, Crystal Structure of Fumarylacetoacetate Hydrolase Complexed With Fumarate And Acetoacetate	191	4	10	46.4			gi8569274

	Multistep	Glutamate-ammonia ligase	187	5	15	42.0			gi2144563

	HPLC	Glutamine synthetase	187	5	15	42.1			gi31982332

	Multistep	Long-chain acyl-CoA dehydrog-enase	122	3	6	48.0			gi726095

10	HPLC	Arginase 1	232	4	13	34.8	0	0	gi7106255

	Multistep		314	6	23		0	0	

	HPLC	Aldo-keto reductase family 1, member D1	120	3	7	37.4			gi20302063

	Multistep		69	4	9				

	HPLC	rCG27878	120	3	7	37.4			gi149065268

	Multistep		69	4	9				

11	HPLC	Ornithine transcarbamyl-ase, isoform CRA_f	298	5	14	42.0	1	14.29	gi148703731

	Multistep		285	7	19		0	0	

	HPLC	Ornithine transcarbamyl-ase	297	5	14	39.8			gi762985

	Multistep		284	7	20				

	HPLC	Otc protein	297	5	14	39.3			gi19353187

	Multistep		284	7	20				

12	HPLC	Alcohol dehydrogenase 1 (class I)	156	3	9	39.7	0	0	gi6724311

	Multistep		102	3	9		0	0	

	HPLC	Alcohol dehydrogenase 1 (class I), isoform CRA_b	156	3	11	35.3			gi148680154

	Multistep		102	3	11				

	HPLC	Electron transferring flavoprotein, alpha polypeptide	155	3	7	35.0			gi13097375

	Multistep		429	9	31				

	HPLC	MAWD binding protein homolog 1	139	5	21	32.0			gi31560132

13	Multistep	Chain A, Methyl-transferase	284	5	18	32.4	1	12.5	gi1942407

	HPLC		236	4	19		0	0	

	Multistep	Glycine N-methyl-transferase	284	5	18	32.4			gi6754026

	HPLC		236	4	19				

	Multistep	Electron transferring flavoprotein, alpha polypeptide	121	2	7	35.0			gi13097375

14	HPLC	Glutathione S-transferase mu 1	193	5	19	26.0	1	25	gi6754084

	Multistep		237	6	24		0	0	

	HPLC	mCG131602, isoform CRA_a	193	5	17	29.1			gi148669989

	Multistep		237	5	21				

	HPLC	mCG131602, isoform CRA_b	193	5	19	26.6			gi148669990

	Multistep		237	5	24				

	HPLC	mCG131602, isoform CRA_c	193	5	19	26.7			gi148669991

	Multistep		237	5	24				

15	HPLC	Hemoglobin beta	378	6	47	15.7	0	0	gi229301

	Multistep		403	7	52		0	0	

	HPLC	Hemoglobin, beta adult major chain	378	6	47	15.7			gi31982300

	Multistep		403	7	52				

	HPLC	Delta-globin	219	4	29	16.0			gi122717

	Multistep		208	5	35				

16	HPLC	Fatty acid binding protein 1, liver	149	3	22	14.2	0	0	gi8393343

	Multistep		115	3	22		0	0	

## Discussion

A comparison of data-dependent MS/MS fragmentation within the proposed direct inject multi-step small range parent ion MS scan and a direct inject single step full range parent ion MS scan were conducted on selected 2D gel protein spots and the results were compared with the more traditional HPLC MS/MS method. The only difference between the two direct inject methods were the scan range before the data-dependent MS/MS fragmentation; the former one (multi-step) utilized 4 segments of overlapping narrow range parent ion MS scans followed by daughter ion MS/MS scans, while the latter one (full range) utilized a full range parent ion MS scan (400-2000 m/z) followed by daughter ion MS/MS scans. The MOWSE scores of the multi-step small range scan were greater than those of full range scan method due to discontinuous ion suppression leading to fewer candidate parent ions. The results of the multi-step small range scan was comparable with the results of the HPLC method (data not shown). In the HPLC method, due to the variance of peak width for the eluting peptides, it is not feasible to adjust the time used for each segment in the MS scan of each parent ion. Some peaks lose a great degree of information when the multisegment approach is employed, particularly the moderate to low abundance peaks. The use of direct injection with continuous, stable parent ion composition makes the multisegment parent ion scanning feasible.

As described above, the multistep direct inject nanoESI/MS/MS method yielded at a minimum comparable, and in most cases greater, confidence in the correct identification of proteins from a polyacrylamide gel matrix. The 16 spots analyzed covered a wide mass range, from 14,200 to 164,000 amu including the low, medium, and high abundance levels indicating that the multistep direct inject method is robust for protein identification. The advantages of this chip-based nanoESI/MS/MS method include fully automated, lack of cross-contamination, and high throughput with sensitivity sufficient to identify multiple proteins per sample. The number of segments, mass range and running time for each segment may be optimized to accommodate the complexity of different matrixes.

Mass spectrometry methods have been recognized as a general strategy for protein characterization. Although HPLC-MS/MS is commonly utilized for protein identification [8] it has the disadvantage of a lengthy run time, high cost per sample due to instrument time, and possible sample cross-contamination due to column carryover. Here we introduce a novel 4-segment multistage direct inject nanoESI LTQ-FT-ICR-MS/MS method that can be used to analyze a proteolytic digest within approximately 3 minutes. Multiple overlapping narrow range parent ion mass scans followed by iterative MS/MS can effectively minimize the discontinuous ion suppression observed with broad range mass spectral windows (400-2500 m/z) common in other direct inject nanoESI-MS/MS methods for protein identification. The current method is robust with wide mass range coverage and multiple protein identification in samples. While an LTQ-FT-ICR-MS was utilized for the current comparison, high mass accuracy and resolution is not a requirement of the method. Indeed, as MS/MS agreement with fragmentation patterns is the basis of peptide identification, the method is amenable to use on a variety of mass spectrometry platforms which provide sufficient parent ion resolution for selection of multiple peptide features for fragmentation--ie all commercially available mass spectrometry platforms currently used for proteomics workflows. As this method can substitute for the traditional HPLC-MS/MS method for the protein analysis of 2D gel spots with a much lower cost, we propose that the current practice of identification of protein spots of interest should change to an analysis of the entire population of proteins present on 2D gels to enhance the understanding of the population represented within an experiment.

## Conclusion

A multi-step, chip-based nanoESI-MS/MS method is proposed for protein identification. This method consists of 4 segments with overlapping narrow range scans followed by data-dependent MS/MS fragmentation. With a narrow range scan, a greater number of moderate to low abundance ion features are found than when a full experimental range parent ion scan is utilized. The advantages of this method include full automation, lack of cross-contamination, low cost, and high throughput. It should be noted that the cost of the direct inject platform (Advion Nanomate) and associated disposables (tips, chip) is analogous to a moderate cost HPLC system.

## Materials and methods

All reagents and solvents for mass spectrometry of LC/MS grade were obtained from Fisher Scientific (Waltham, MA, USA). Modified trypsin was purchased from Promega (Madison, WI, USA) or Sigma-Aldrich (St. Louis, MO, USA). ZipTips_C18 _was purchased from Millipore (Bedford, MA, USA).

### 2D-SDS-PAGE of Liver Proteins

Adult C57BL/6J mice were purchased from The Jackson Laboratory (Bar Harbor, ME). Animals were housed by the University of Louisville in a dedicated room at 22°C, with a 12 hour alternating light/dark cycle, and were maintained on Purina LabDiet #5015 and water *ad libitum*. The University of Louisville is an 'Association for Assessment and Accreditation of Laboratory Animal Care' (AAALAC)-approved facility. Rat liver was homogenized on ice in 7 M urea, 2 M thiourea with 4% CHAPS detergent. 500 ug liver protein (measured by the Bradford method) was separated by isoelectric focusing with non-linear, pH 3-10, 180 mm × 3 mm × 0.5 mm Immobiline DryStrips (GE Healthcare, Piscataway, NJ) according to the manufacturer's instructions. Passive rehydration was accomplished in a 7 M urea, 2 M thiourea, and 4% CHAPS solution containing 2.8 mg/ml DTT and 2% pH 3-10 IPG buffer. The proteins were focused for 28,000 Vhrs. Before transfer of proteins for second dimension separation according to molecular weight, the strips were equilibrated first with 3.5 mg/mL DTT contained in equilibration buffer (1.5 M Tris pH 8.8 buffer containing 6 M urea, 34.5% glycerol, and 2% SDS) for 30 minutes and second with 45 mg/ml iodoacetamide in equilibration buffer for an additional 30 minutes. The strips were then loaded onto 12% acrylamide gels and separated at 70 volts for 24 hours at room temperature. Gels were stained with colloidal Coomassie Blue G-250 and washed in deionized water for at least 48 hours. Gel images were collected using an Epson Expression 1680 desktop scanner with transparency adapter at 266 dpi.

### Proteolytic Digestion of Selected Protein Spots

Selected spots representing high and low molecular weight, intense and faint protein expression were excised from 2D-SDS-PAGE gels and destained with 50% ethanol (EtOH) and 50 mM NH_4_HCO_3 _at room temperature with a minimum of 5 washes. The gel pieces were dehydrated in ethanol and subjected to in-gel protease digestion with modified trypsin (10 mg/μL, Promega) in 50 mM NH_4_HCO_3 _for 18 hours. Tryptic peptides were extracted via sequential steps of 50% EtOH in 0.1% formic acid followed by 95% EtOH in 0.1% formic acid. The extracted peptides were split into 2 aliquots, desalted with C18 ziptips (Millipore, Bedford, MA, USA), lyophilized, and stored at -80°C until analysis.

### LC-nano-ESI-MS/MS

The extracted peptides were dried and resolubilized in 30 μL of 3% acetonitrile (ACN), 97% water, 0.1% formic acid and transferred to auto-sampler vials for LC-MS/MS analysis. The binary gradient elution mobile phase initially consisted of 90% A: 10% B. A was 3% acetonitrile (ACN) and 0.1% formic acid in water, and B was 97% ACN and 0.1% formic acid in water. Mobile phase A at 90% was maintained for the first 6 min and decreased linearly to 45% over 10 min, maintained for 0.1 min and then changed to 10% and maintained for 2 min. Finally, the mobile phase A was increased to 90% and the column was re-equilibrated for a further 8 min. Sample temperature was maintained at 10°C in the autosampler prior to analysis. The flow-rate was 10 μL/min and split at approximately a 1:10 ratio before introduction to the mass spectrometry system.

An LTQ-FT-ICR MS system (Thermo-Electron, Waltham, MA, USA) was utilized for data acquisition. The mass analysis method consisted of one segment with three scan events with an initial delay of 2 min following injection and sample spray initiation. The 1^st ^scan event was a broad range FT-ICR-MS scan (400-1200 m/z) with 100,000 resolution for parent ion selection followed by 2 other scan events with data dependent MS/MS for fragmentation of the 2 most intense ions with +2 or +3 charges. Unassigned, +1, and +4 charges were rejected. The ion fragmentation process was conducted in the ion trap (35 eV CID energy) with a parent mass feature isolation width of 2 m/z. Dynamic exclusion to prevent multiple fragmentation events with same parent mass feature utilized a m/z window of 0.5-1.5 m/z; repeat and exclusion windows of 15 s and 30 s and an exclusion list size of 48. The total acquisition time for the HPLC-ESI-MS/MS method was 26 min.

### Multi-step direct inject nano-ESI-FT-ICR-MS/MS

An aliquot (7 μL) of the liver protein spot tryptic digest was introduced via chip-based nanoelectrospray with an Advion TriVersa Nanomate interfaced to the LTQ-FT-ICR-MS. This chip-based nanoESI-MS/MS method consisted of a prescan (400-2000 m/z) followed by 4 segments overlapping narrow range scans (400-600, 550-750, 700-950, and 900-1200 amu; 50 amu overlap) with data-dependent MS/MS fragmentation of selected parent ions of the top most intense +2 or +3 peaks in each segment. The top 10 most intense +2 or +3 peaks were subjected to CID fragmentation from each segment. For each segment, a total of 11 scan events were included: an initial FT-ICR parent mass scan with 100,000 resolution followed by 10 other scan events with data dependent MS/MS of +2 or +3 charged ions. These 11 scan events were cycled until the segment time was exceeded. Unassigned, +1, and +4 charges were rejected. Parent mass fragmentation was initiated in the ion trap (35 eV CID energy) with an ion isolation width of 2 m/z. Dynamic exclusion was employed with a mass width 0.5-1.5 m/z, repeat and exclusion duration are 180.00 s with exclusion list size of 500. Scan times for the full rang and the narrow mass ranges are both 500 microseconds while the scan time for MS/MS is 100 microseconds. The sample spray characteristics were stable (greater than 10 minutes) with ion current between 10-90 nA (1.5 kV spray voltage and 0.10 psi head pressure). The duration for the 4 segments were 1.4, 1.0, 0.4, and 0.2 minutes, respectively, with a total acquisition time of 3 minutes.

### Peptide identification

Common to the two workflows above, peptides were identified by NCBInr database searching with an in-house Mascot Server (Matrix Scientific). Parameters for the searches were: Mammalian proteins, ± 0.8 daltons for the parent peptide, ± 0.4 daltons for fragmentation masses, 2 missed trypsin cleavage sites allowed, and carbamidomethylation of cysteins as a variable modification. Acceptable protein identifications required a Mascot MOWSE score greater than 65 and a minimum of 2 peptides.

## Competing interests

The authors declare that they have no competing interests.

## Authors' contributions

JC and REN planned the experiments; LC was responsible for the preparation of 2D gel spot samples; JC carried out the experiments, performed data analysis and wrote the first draft of the manuscript; LC and REN critically reviewed the content of the manuscript before submission. All authors read and approved the final manuscript.
